# Vanishing Bile Duct Syndrome: A Differential Diagnosis for Painless Jaundice

**DOI:** 10.7759/cureus.68724

**Published:** 2024-09-05

**Authors:** Siona Sabu, Gayatri Chakrabarty, Fatima Shahid

**Affiliations:** 1 Internal Medicine, Surrey and Sussex Healthcare NHS Trust, Redhill, GBR; 2 Gastroenterology and Hepatology, Surrey and Sussex Healthcare NHS Trust, Redhill, GBR; 3 Gastroenterology, Surrey and Sussex Healthcare NHS Trust, Redhill, GBR

**Keywords:** cholestasis, ductopenia, hodgkin’s lymphoma, painless jaundice, vanishing bile duct

## Abstract

Vanishing bile duct syndrome (VBDS) is a clinicopathological term coined to describe an acquired liver disease characterised by progressive destruction and disappearance of intrahepatic biliary ducts. We report the case of a 69-year-old female who presented with painless jaundice, fatigue, and weight loss. Initial blood workup revealed hyperbilirubinemia, transaminitis, elevated alkaline phosphatase, and a raised international normalised ratio. Para-aortic lymphadenopathy on computed tomography of the abdomen was biopsied to confirm the diagnosis of Hodgkin’s lymphoma. Vanishing bile duct syndrome is a paraneoplastic phenomenon of Hodgkin’s lymphoma, a recognised cause of cholestatic jaundice, and our patient’s liver biopsy was diagnostic of the same. Despite treatment with steroids, ursodeoxycholic acid, and chemotherapy, our patient passed away. This case report aims to highlight VBDS as a differential diagnosis for painless jaundice in the context of cholestatic liver dysfunction. We believe reporting such cases irrespective of their outcome will help raise awareness of VBDS among clinicians, thus bettering the rate at which it is diagnosed and treated, thereby improving patient outcomes.

## Introduction

Vanishing bile duct syndrome (VBDS) is a histological diagnosis of an acquired cholestatic liver disease. It is marked by loss of interlobular bile ducts in more than 50% of portal tracts if at least 10 portal tracts are examined. Although the underlying pathogenesis is not well established, VBDS has been associated with a variety of causes, including infections, drugs, autoimmune diseases, and malignancies such as Hodgkin’s lymphoma. If not treated, it can progress to biliary cirrhosis, liver failure, and death. Management primarily focuses on treating the underlying disease or removing the offending agent alongside supportive symptomatic care [1-3]. We report a case of Hodgkin’s lymphoma presenting for the first time as VBDS.

## Case presentation

A 69-year-old female patient presented with a three-week history of jaundice and a two-month history of fatigue and unintentional weight loss. Jaundice was gradually progressive and was not associated with any colour changes in her urine or stool. She denied abdominal pain, fevers, night sweats, nausea, vomiting, and diarrhoea. Fatigue had progressed to a point where the patient who was previously independent of her activities of daily living was struggling with the same at the time of admission. She also reported a 13-kg unintentional weight loss over two months. The patient denied joint pains, joint swelling, skin rash, oral ulcers, hair loss, or other symptoms of autoimmune disease. There was no history of preceding viral illness, recent surgery, or travel.

Past medical history included interstitial lung disease, pulmonary tuberculosis as a child (treatment details were not available), hypertension, and hypercholesterolemia. Socially, the patient did not consume alcohol. Her regular medications included bisoprolol 1.25 mg OD and famotidine 20 mg OD, neither of which were started in the preceding six weeks.

Physical examination at the time of admission revealed scleral icterus and jaundice. No hepatic flap or other stigmata of chronic liver disease were noted. No cervical, axillary, or inguinal lymphadenopathy was appreciated on examination. Initial blood workup noted raised inflammatory markers (C-reactive protein of 27 mg/L) and lymphopenia (0.6 × 10^9^/L). Admission liver function tests were notable for a total bilirubin of 370 µmol/L, alkaline phosphatase of 575 IU/L, alanine aminotransferase of 93 IU/L, aspartate aminotransferase of 135 IU/L, albumin of 25 g/L, and the international normalised ratio of 1.6. Blood tests at admission and subsequent non-invasive liver screens are presented in Table 1.

**Table 1:** Admission blood tests and non-invasive liver screen.

Investigation	Result (normal range)
Haemoglobin	130 (120–150 g/L)
White blood cell count	5.8 (3.7–11.1 × 10^9^/L)
Platelet count	278 (150–400 × 10^9^/L)
Haematocrit level	0.38 (0.36–0.46)
Mean cell volume	86 (82–98 fL)
Red blood cell distribution width	23.9 (9.9–15.5%)
Mean cell haemoglobin level	28.7 (27.3–32.6 pg)
Mean cell haemoglobin concentration	335 (320–350 g/L)
Neutrophil count	4.1 (1.5–7.4 × 10^9^/L)
Lymphocyte count	0.6 (1.1–4 × 10^9^/L)
Monocyte count	0.8 (0–0.95 × 10^9^/L)
Eosinophil count	0.3 (0–0.7 × 10^9^/L)
Basophil count	0.1 (0–0.2 × 10^9^/L)
Prothrombin time	20.6 (11.6–14.9s)
International normalised ratio	1.6 (0.8–1.1)
Activated partial thromboplastin time	36.6 (26.7–33.8)
Activated partial thromboplastin time ratio	1.17 (0.88–1.12)
Sodium level	136 (136–145 mmol/L)
Potassium level	3.7 (3.5–5.1 mmol/L)
Urea level	3.1 (2.1–7.1 mmol/L)
Enzymatic creatinine	61 (45–84 µmol/L)
Calcium level	2.19 (2.15–2.55 mmol/L)
Adjusted calcium level	2.49 (2.15–2.55 mmol/L)
Inorganic phosphate level	0.92 (0.91–1.45 mmol/L)
Total bilirubin level	370 (0–21 µmol/L)
Alanine aminotransferase	93 (0–33 IU/L)
Aspartate aminotransferase	135 (0–32 IU/L)
Alkaline phosphatase	575 (35–104 IU/L)
Gamma-glutamyl transpeptidase	155 (6–42 IU/L)
Albumin level	25 (35–52 g/L)
Hepatitis A IgM antibody	Not detected
Hepatitis B surface antigen	Not detected
Hepatitis B core total antibody	Not detected
Hepatitis C antibody	Not detected
Hepatitis E virus IgM	Not detected
Hepatitis E virus IgG	Not detected
Cytomegalovirus IgG antibody	Detected
Cyclic citrullinated peptide antibodies	1.4 (0–7 IU/mL)
Anti-nuclear antibody panel	0.2 (0–0.9)
Double-stranded DNA antibody	1 (0–9 IU/mL)
Anti-mitochondrial antibody	Negative
Liver kidney microsomal antibody	Negative
Smooth muscle antibody	Negative
Gastric parietal cell antibody	Negative
IgG	12.2 (6–16 g/L)
IgA	3.32 (0.8–4 g/L)
IgM	0.27 (0.5–2 g/L)
C-reactive protein level	27 (0–5 mg/L)

**Figure 1:**
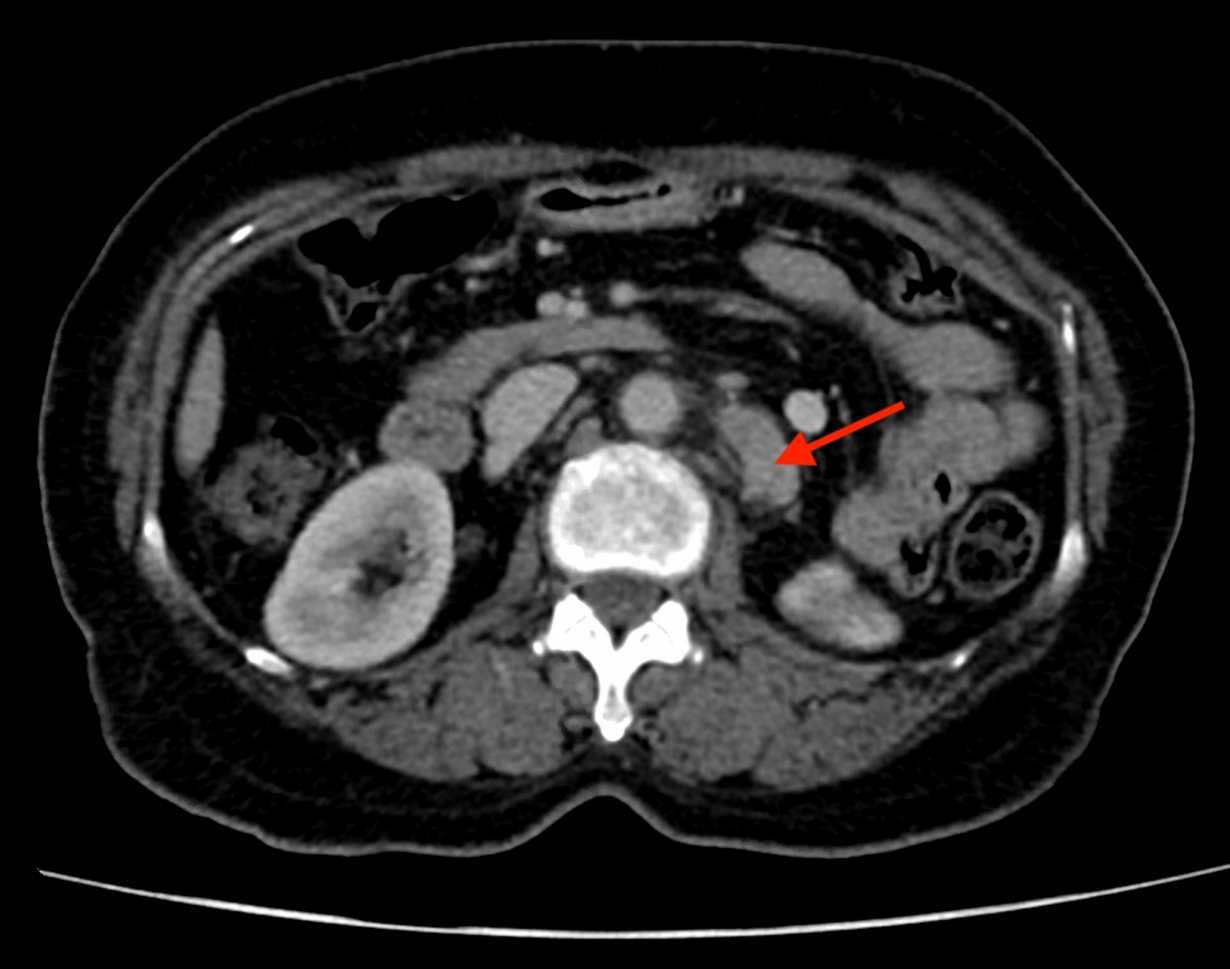
Para-aortic lymphadenopathy on contrast-enhanced computed tomography of the abdomen and pelvis (axial view).

**Figure 2:**
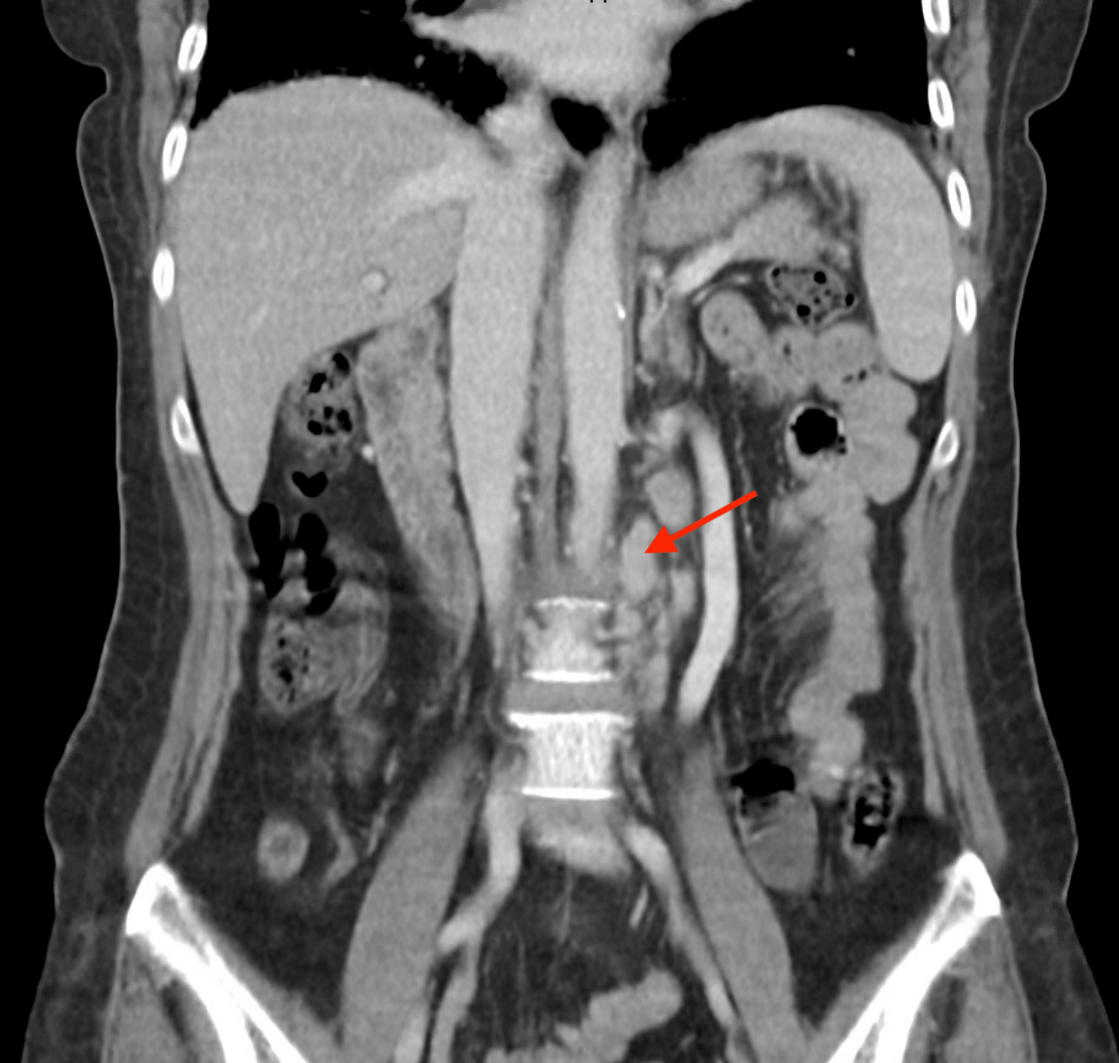
Para-aortic lymphadenopathy on contrast-enhanced computed tomography of the abdomen and pelvis (coronal view).

Initial computed tomography of the abdomen and pelvis showed para-aortic lymphadenopathy and no intra or extrahepatic biliary duct dilatation (Figures 1, 2). This was confirmed on magnetic resonance cholangiopancreatography which also noted multiple bony lesions suspicious for metastases.

**Figure 3:**
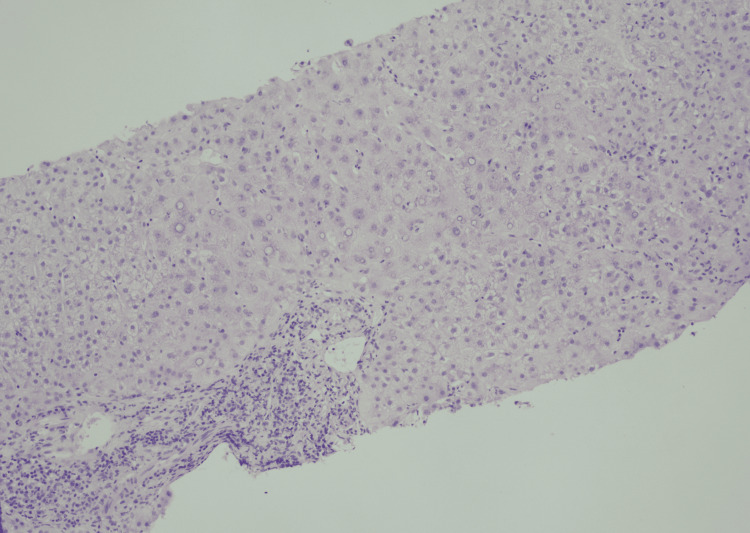
Lobular inflammation and portal tract lacking small bile ducts (×100 hematoxylin and eosin).

**Figure 4:**
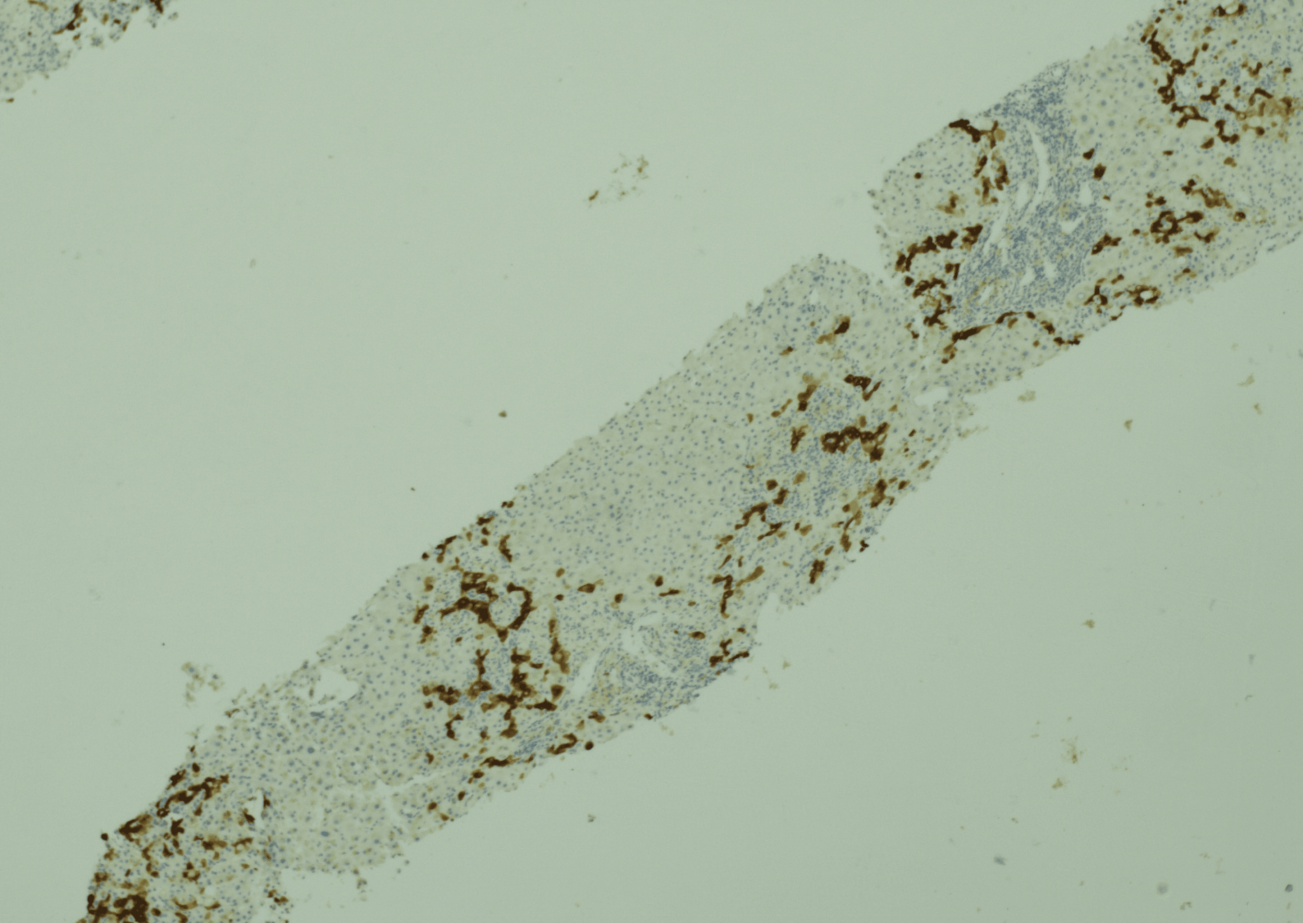
Cytokeratin 7 stain demonstrating a lack of bile ducts and prominent biliary metaplasia of hepatocytes (×40).

A para-aortic lymph node biopsy confirmed classical Hodgkin’s lymphoma of mixed cellular subtype. The bony lesions noted earlier were attributed to osseous metastases from Hodgkin’s lymphoma itself following further investigation with a magnetic resonance imaging scan of the spine and a contrast-enhanced computed tomography of the chest, thus completing a staging scan. A liver biopsy showed lobular inflammation and the portal tract lacking small bile ducts (Figure 3). Cytokeratin 7 stain demonstrated a lack of bile ducts and prominent biliary metaplasia of hepatocytes (Figure 4). Severe cholestasis and ductopenia were highly suggestive of VBDS.

The patient was started on ursodeoxycholic acid at 13 mg/kg after imaging ruled out any obstructive pathology. While awaiting liver biopsy results, high-dose steroids (prednisolone 60 mg OD) and allopurinol were commenced in response to the confirmation of Hodgkin’s lymphoma. With liver biopsy results in keeping with VBDS, a rare but recognised paraneoplastic phenomenon of Hodgkin’s lymphoma, chemotherapy was initiated. Given the severe hepatic dysfunction, renally metabolised drugs, gemcitabine and oxaliplatin were the chemotherapy agents of choice. Although the total bilirubin level improved shortly after the commencement of chemotherapy, the patient deteriorated clinically and eventually succumbed to neutropenic sepsis.

## Discussion

VBDS is a clinicopathological term coined to describe an acquired liver disease characterised by progressive destruction and disappearance of intrahepatic biliary ducts (ductopenia). It is a result of multiple insults to the liver eventually leading to cholestatic hepatitis, cirrhosis, and, finally, liver failure [1].

Aetiologies associated with VBDS include various infections, malignancies, immune-mediated conditions, hepatic ischemia, and several drugs [2,3]. The underlying pathophysiology is yet to be fully understood; however, the various theories postulated include biliary duct destruction mediated by antibodies, cytotoxic effects of T cells, and high levels of cytokines released by the tumour causing bile duct epithelial cell destruction [4-6].

Patients with VBDS typically present with jaundice, fatigue, weight loss, abdominal pain, and persistent pruritus. Cholestasis may also be evidenced by the formation of gallstones, dyslipidaemias, malabsorption, xanthelasmas, and deficiencies of fat-soluble vitamins [3,7]. As acute cholestatic hepatitis is the hallmark of VBDS, persistent elevations of alkaline phosphatase and bilirubin are also seen [7].

Our patient presented with cholestatic liver injury and para-aortic lymphadenopathy on computed tomography of the abdomen, biopsies of which established a diagnosis of Hodgkin’s lymphoma. Cholestatic dysfunction as an initial presentation of classical Hodgkin’s lymphoma is very rarely seen, accounting for less than 4% of cases [8]. Malignancy-related causes of cholestasis in such patients could be lymphomatous infiltration of the liver itself, obstruction of bile flow due to tumour mass effect, malignancy-associated immunodeficiency causing reactivation of a particular virus, or paraneoplastic processes [9].

Investigations including serological tests and imaging were aimed at looking for common causes of cholestasis as well as the above-mentioned cancer-related causes of cholestasis. Common intrahepatic causes of cholestasis such as primary biliary cirrhosis and primary sclerosing cholangitis were tested for with an autoimmune serological panel and common extrahepatic causes such as choledocholithiasis, bile duct strictures, and tumours by a magnetic resonance cholangiopancreatography (an endoscopic retrograde cholangiopancreatography is a suitable choice of investigation as well). A liver biopsy showing the loss of interlobular bile ducts in more than 50% of portal tracts given that a minimum of 10 portal tracts visualised in a specimen is diagnostic of VBDS [1,3,10].

The treatment of VBDS centres around managing the causative process itself. The aggressive nature of VBDS with rapid progression from severe cholestasis to liver failure and death warrants early assessment for consideration of liver transplantation [7]. Due to the rarity of the disease and the paucity of case reports, the ideal treatment strategy for VBDS related to Hodgkin’s lymphoma is not established yet. The biggest challenge around its treatment is that these patients usually have severe liver dysfunction, and first-line agents recognised for the management of Hodgkin’s lymphoma are associated with hepatotoxicity as they are metabolised by the liver. However, multiple reports have been published highlighting remission of lymphoma and resolution of VBDS with the standard-of-care ABVD (doxorubicin, bleomycin, vinblastine, dacarbazine) regimen as well as modified chemotherapy regimens. Various modified treatment approaches reported include dose reduction of drugs that are cleared hepatically as well as standard chemotherapy regimens being preceded by various therapies to reduce lymphoma burden [2,6,10-14]. Wong et al. also reported treatment with ABVD chemotherapy, followed by autologous hematopoietic cell transplantation resulting in complete remission of Hodgkin’s lymphoma and regeneration of interlobular bile ducts on post-treatment liver biopsy [15]. Our patient was treated with ursodeoxycholic acid, a hydrophilic dihydroxy bile acid, and steroids to reduce VBDS-related cholestasis before starting a modified chemotherapy regimen with renally cleared drugs. Although she did not have a desirable outcome, success stories have been reported following similar treatment strategies [2,10,13].

## Conclusions

In summary, VBDS is a well-recognised but rare paraneoplastic phenomenon of Hodgkin’s lymphoma. It is key for clinicians to have a high index of suspicion when reviewing patients with painless jaundice in the context of hyperbilirubinemia and features of cholestasis to suspect Hodgkin’s lymphoma-associated VBDS. Ultimately VBDS-related Hodgkin’s lymphoma is a diagnosis essentially of exclusion. A liver biopsy confirming diagnostic findings of cholestasis along with ductopenia having ruled out malignant hepatic involvement and other known causes of liver dysfunction would point towards VBDS. Early involvement of a multidisciplinary team and focusing on treatment with appropriate chemotherapy/radiotherapy tailored to each patient are the fundamental principles of managing patients with VBDS in the setting of Hodgkin’s lymphoma.
